# Supplementary material for: MGST1 Protects Pancreatic Ductal Cells from Inflammatory Damage in Acute Pancreatitis by Inhibiting Ferroptosis: Bioinformatics Analysis with Experimental Validation
Source: Int J Mol Sci. 2025 Feb 22;26(5):1899. doi: 10.3390/ijms26051899 (PMC11899814; doi:10.3390/ijms26051899)
Supplement: Supplementary file 1 [file ijms-26-01899-s001.zip › ijms-3453707-supplementary.pdf]

Table S1. The primers used in the RT-qPCR

Gene	Sequence（5‘-3’）
Human-AQP3	Forward Primer: GGGGAGATGCTCCACATCC<br>Reverse Primer: AAAGGCCAGGTTGATGGTGAG
Mouse-AQP3	Forward Primer: CGAAGTGCCAGATTGCATCATAA<br>Reverse Primer: AGATGCTCCACATCCGCTAC
Human-TRIB2	Forward Primer: ATGAACATACACAGGTCTACCCC<br>Reverse Primer: GGGCTGAAACTCTGGCTGG
Mouse-TRIB2	Forward Primer: GACTCCGAACTTGTCGCATTG<br>Reverse Primer: GGCACGAAAAACGTGGTCT
Human-MGST1	Forward Primer: ATGACAGAGTAGAACGTGTACGC<br>Reverse Primer: TACAGGAGGCCAATTCCAAGA
Mouse-MGST1	Forward Primer: CGCATTCCAGAGGATAACCAA<br>Reverse Primer: CAAAGCCAGCGCAGTCTTCT
IL6	Forward Primer: CCTGAACCTTCCAAAGATGGC<br>Reverse Primer: TTCACCAGGCAAGTCTCCTCA
IL1β	Forward Primer: ATGATGGCTTATTACAGTGGCAA<br>Reverse Primer: GTCGGAGATTCGTAGCTGGA
IL8	Forward Primer: TTTTGCCAAGGAGTGCTAAAGA<br>Reverse Primer: AACCCTCTGCACCCAGTTTTC
IL18	Forward Primer: TCTTCATTGACCAAGGAAATCGG<br>Reverse Primer: TCCGGGGTGCATTATCTCTAC
ACSL4	Forward Primer: CATCCCTGGAGCAGATACTCT<br>Reverse Primer: TCACTTAGGATTTCCCTGGTCC
GPX4	Forward Primer: GAGGCAAGACCGAAGTAAACTAC<br>Reverse Primer: CCGAACTGGTTACACGGGAA
NRF2	Forward Primer: TCAGCGACGGAAAGAGTATGA<br>Reverse Primer: CCACTGGTTTCTGACTGGATGT
GAPDH	Forward Primer: ACAACTTTGGTATCGTGGAAGG<br>Reverse Primer: GCCATCACGCCACAGTTTC
